# Sedentary Behavior among 6–14-Year-Old Children during the COVID-19 Lockdown and Its Relation to Physical and Mental Health

**DOI:** 10.3390/healthcare9060756

**Published:** 2021-06-18

**Authors:** Rima Breidokienė, Roma Jusienė, Vaidotas Urbonas, Rūta Praninskienė, Sigita Girdzijauskienė

**Affiliations:** 1 Institute of Psychology, Faculty of Philosophy, Vilnius University, 01513 Vilnius, Lithuania; roma.jusiene@fsf.vu.lt (R.J.); sigita.girdzijauskiene@fsf.vu.lt (S.G.); 2 Clinic of Children’s Diseases, Institute of Clinical Medicine, Faculty of Medicine, Vilnius University, 01513 Vilnius, Lithuania; vaidotas.urbonas@mf.vu.lt (V.U.); ruta.praninskiene@mf.vu.lt (R.P.)

**Keywords:** sedentary behavior, physical activity, screen time, COVID-19

## Abstract

As a result of the outbreak of SARS-CoV-2 and consequent restrictions in spring 2020, children in many countries might be engaged in more sedentary behavior and have limited possibilities to access the necessary level of physical activity to maintain their physical and mental health. The aim of this study was to explore the relationships between child sedentary behavior, physical activity, mental and physical health, and parental distress in a sample of Lithuanian children aged 6–14 years during the COVID-19 pandemic lockdown in March–June 2020. Parents of 306 children (52.9% female) completed an online survey in May–June 2020 and reported on their children’s screen time for educational and recreational (leisure) purposes, the level of physical activity and time outdoors, somatic symptoms, and emotional well-being and behavior. Parents also reported on stressful life events in the family and personal distress. The results revealed that 57.5% of children exceeded the recommended maximum of 2 h of recreational screen time per day, and 33.6% of the children did not meet the recommended guidelines of 60 min of physical activity per day. Longer screen time for educational purposes and parental distress significantly predicted a higher prevalence of somatic symptoms in children and parental distress also served as a significant predictor of children’s decreased emotional well-being and behavior. These results highlight the importance of psychosocial support interventions for parents who experience distress when raising children at a stressful time, such as during a pandemic.

## 1. Introduction

The World Health Organization (WHO) declared a pandemic for the new coronavirus SARS-CoV-2 (which causes COVID-19) on 11 March 2020. The Lithuanian Government ordered a national lockdown on 16 March. To prevent the spread of COVID-19 infection in kindergartens and primary and secondary schools, distance education was introduced on 16 March and continued till 16 June. The second lockdown was introduced in November 2020 and is still going. The social and physical restrictions of the lockdown affected around half a million children aged 0–18 years in Lithuania. All these restrictions compromised the children’s health-related behavior and everyday routines. As a result, children were more engaged in sedentary behavior (SB) and may have had limited possibilities to maintain the necessary level of physical activity (PA) to preserve their physical and mental health. 

Sedentary behavior is defined as any waking behavior with an energy expenditure of ≤1.5 metabolic equivalents while in a sitting or reclining posture [1]. Excessive sedentary behavior is widely recognized to have a negative health effect [2,3], yet a systematic review of the relationships between the different types of SB and health indicators in preschool and school-aged children established screen time as the culprit for most detrimental effects [4,5]. According to sedentary behavior guidelines, for children aged 2, sedentary screen time should not exceed 1 h per day, and for 5–17-year-olds, the recommended recreational screen time is no more than 2 h per day [6]. However, before the pandemic, a lot of Lithuanian children failed to meet the sedentary behavior recommendations for their age group [7,8,9].

Since the onset of the COVID-19 pandemic, a significant increase in exposure to screen time has been reported all over the world, including Lithuania [10,11,12,13,14].

A systematic review of 235 studies examining the relationship between SB and health indicators in children aged 5–17 years documented SB as a risk factor for cardiometabolic diseases, behavior problems, obesity, and even self-esteem [4]. SB leads to obesity in children and adolescents through increased eating while viewing a screen [15,16] and less desirable food choices, particularly in overweight children [17,18].

Sedentary behavior is closely related to physical activity [19], though these concepts are not synonymous or overlapping [20]. A decrease in the level of physical activity is associated with numerous negative health effects throughout the life span [21]. For 5–17-year-olds, 60 min of moderate-to-vigorous physical activity per day is recommended, including bone-loading and muscle-strengthening activities at least three times per week; also, several hours of light physical activity (e.g., walking or playing) should be practiced daily [6].

Since the onset of the COVID-19 pandemic, a considerable decrease in the level of children’s physical activity was found in many countries [11,12,13,14,18,22,23]. 

SB and screen time are associated with an increased risk for poorer mental health outcomes [24,25]. Overall, the COVID-19 pandemic has negatively affected children’s mental health: the recent studies revealed high rates of psychological distress, anxiety, depression, difficulty with concentrating, and post-traumatic symptoms among children of different ages and in various socioeconomic contexts [14,26,27]. The impact of the lockdown reinforces the need to identify the risk factors for physical and mental health problems in children during the COVID-19 pandemic. Such factors could be high rates of SB and low rates of PA [28,29]. Despite the established links between emotional problems, SB, and PA, it is not known whether similar results would be found in younger children and whether these factors could help recognize children at risk of maladaptive functioning in stressful times.

Moreover, different types of sedentary behavior may have a different, not only negative, impact on children’s functioning, e.g., reading vs. screen-use time [4]. Screen use for leisure purposes is sometimes predicted to produce the most detrimental effect on children’s mental health. Moreover, further analysis is needed to understand the parental influence on children’s SB and PA, as they play a major role in establishing routine and screen-time limits. Parents serve as role models for children’s healthy behavior and implement rules limiting their screen time and engaging in physical activity. One of the significant parental variables that needs to be considered when analyzing the effect of SB on children’s physical and mental health is parental mental health. Parental mental health problems (e.g., parenting, financial or life event stress, and anxiety or depression symptoms) may compromise parental interactions with children and lead to unhealthy behavior in children [30]. Elevated levels of stress may also render parents less responsive to children’s physical and emotional needs [31] and less prone to regulate their own and their children’s screen time and physical activity [32,33].

The global COVID-19 pandemic is a significant stressor for the whole family. Dealing with the lockdown during the pandemic is a stressful experience for many parents who must balance work, raising children, and personal life without much outside help [34,35,36,37,38,39]. Few data exist about the relationship between children’s sedentary behavior and physical and mental health in the context of the acute threat of the COVID-19 pandemic for parental mental health. Therefore, we need to take into consideration the factor of parental mental health when examining the relationship between SB and physical and mental health in children during the COVID-19 confinement.

It is clear that COVID induced a significant decrease in children’s PA and an increase in SB, but little is known about how these changes affected social and physical health in the specific context of the strict first lockdown, which was characterized by rapid and unexpected changes and feelings of uncertainty and anxiety. More insight is needed to investigate how typically developing children responded to these unforeseen circumstances.

The aim of the current study was to explore the relationships between child SB, PA, mental and physical health, and parental distress in a sample of Lithuanian children aged 6–14 years during the first COVID-19 pandemic lockdown in March–June 2020. We formulated the following research questions:

(1) How are children’s screen time and physical activity related to their physical health and changes in emotional well-being and behavior during the confinement?
(2) How are the variables of parental sociodemography and distress related to children’s SB, PA, and mental and physical health indicators?
(3) What are the risk factors for children’s somatic symptoms and emotional well-being and behavior during the confinement?

Based on the literature review, we formulated the following hypotheses:

**Hypothesis** **1** **(H1).** *Higher screen time, especially for leisure, and lower PA will be linked with a higher prevalence of somatic symptoms and poorer mental health in children during the COVID-19 lockdown.*

**Hypothesis** **2** **(H2).** *Parental distress will be related to higher SB and lower PA in children and pose a significant risk for children’s somatic symptoms and poorer mental health during the COVID-19 lockdown.*

## 2. Materials and Methods

### 2.1. Participants

The participants were 306 children aged 6–14 years (52.9% female) and their parents (95.0% mothers). The average age of the children in the sample was 9.65 years (SD = 1.94 years).

### 2.2. Procedure

The sample included participants from 3 studies: (1) a longitudinal study on Electronic Media Use and Young Children’s Health (*n* = 42; the study started in 2017; the inclusion criteria for participation in the study were to be a Lithuanian-speaking parent or main caregiver of a child aged 1.5 to 5 years old); (2) a longitudinal study on the Early Development of Self-Regulatory Skills (*n* = 91; the study started in 2009; the main criteria for inclusion into the study were the child’s full-term birth, absence of any inborn abnormalities or disorders, an Apgar score ≥ 7, and mother’s age of at least 18 years); (3) the Distance Education of Children During the COVID-19 Pandemic: Threats and Opportunities from an Ecosystem Perspective study (*n* = 176; conducted in 2020; the inclusion criteria for participation in the study were to be a Lithuanian speaking parent or main caregiver of a child aged 7 to 14 years old). The studied sample was made up of typically developing children of parents whose educational level was a little higher than the average level of the Lithuanian population but corresponded to the main tendencies of educational attainment in Lithuania (for a more detailed description of the sample, see Table 1). Parents of children (ages 6–14 years) were contacted as participants of the longitudinal studies (*n* = 133) or recruited through convenience sampling (*n* = 176) and completed an online survey in May–June 2020 at the end of the first lockdown or right after it. To avoid in-person interactions, potential respondents were invited through various social media platforms (e.g., Facebook) and personal e-mail contacts. The baseline survey took approximately 20 min to complete. Participants had the option to take the survey on their smartphone, tablet, or PC. The two longitudinal studies (Electronic Media Use and Young Children’s Health and Early Development of Self-Regulatory Skills) were conducted with the approval of the Regional Ethics Committee of Biomedical Research. In addition, approval from the Ethical Committee of Psychological Research of Vilnius University (Lithuania) was obtained before the start of the data collection.

### 2.3. Measures

#### 2.3.1. Indicators of Child SB and PA

Parents provided information on screen time by answering separate questions about the amount of time their child had spent during the last two months using screen-based devices on both weekdays and weekends on a scale anchored by the options 1 = no or almost no usage, 2 = approximately 30 min per day, 3 = about 1 h per day, 4 = 2 h per day, 5 = 3 h per day, 6 = 4 h per day, 7 = 5 h per day, and 8 = 6 or more hours per day. Parents were asked to report on their child’s average screen use on a typical weekday and weekend for educational and leisure purposes separately. To assess the average daily screen use, each option was first converted to minutes as follows: 1 = 0 min, 2 = 30 min, 3 = 60 min, 4 = 120 min, 5 = 180 min, 6 = 240 min, 7 = 300 min, and 8 = 360 min. The following formula was used to assess the average daily screen use separately for educational and leisure purposes: (screen use on weekdays (converted to minutes) × 5 days + screen use on weekends (converted to minutes) × 2 days)/7 days.

Child’s physical activity was reported by parents answering the question about how much time per day their child had been physically active during the last two months, anchored by the options almost no physical activity, less than 30 min, 30–60 min, and more than 60 min.

Child’s time outdoors was reported by parents answering the question about how much time per day their child had spent outdoors during the last two months from the options almost no time outdoors, less than 30 min, 30–60 min, and more than 60 min.

#### 2.3.2. Indicators of Child Physical and Mental Health

Each child’s somatic symptoms were measured with a set of 6 items asking about the frequency of nausea, constipation, stomach pains, headaches, and other symptoms in the last two months with four answer options: 1 = never, 2 = sometimes, 3 = often, and 4 = very often. A composite variable of each child’s somatic symptoms was created in which a higher score indicated more somatic symptoms felt by the child in the last two months. The internal consistency of the somatic symptoms items was found to be satisfactory (Cronbach’s alpha = 0.67).

The change in each child’s emotional well-being/behavior was measured by a question asking parents to evaluate how their child’s emotional well-being and behavior had changed in the last two months using five answer options: 1 = decreased, 2 = slightly decreased, 3 = no change, 4 = slightly increased, and 5 = increased. Based on the parents’ answers, the participants were divided into 3 groups: 1 = decreased, 2 = no change, and 3 = increased (see Table 1).

### 2.4. Parental Variables

Parental distress was measured with a set of 6 items asking about the frequency of physical pain or symptoms, sadness/depression, irritability/bad moods, anxiety/distress, sleep problems, and lack of energy during the past two months with five answer options: 1 = almost every day, 2 = two–three times per week, 3 = nearly once every week, 4 = nearly once every month, and 5 = rarely or almost never (Cronbach’s alpha = 0.86). A higher score on the variable indicated a higher level of parental distress.

Stressful life events were measured by parents’ answers to the question regarding whether they or their family had experienced any crises, accidents, or stress within the last year, except for the COVID-19 pandemic.

### 2.5. Statistical Analysis

The SPSS 23.0 software package (IBM SPSS Statistics, Armonk, NY, USA, 2015) was used to analyze the data. The distribution of variables in the groups was calculated using frequency distribution tests. Relationships between the variables were calculated using Spearman’s correlations. Comparisons of means between groups were conducted with the Mann–Whitney test (2 groups), and the chi-square test was used to evaluate differences between categorical or dichotomous variables. Hierarchical multiple linear regression analysis was performed to explore whether the study variables predicted children’s somatic symptoms and hierarchical multinomial logistic regression analysis was conducted to examine the predictors of change in children’s emotional well-being/behavior.

## 3. Results

The results presented in Table 1 show that most parents (73.5%) had university-level education (≥16 years of education; see Table 1). A total of 88.6% were married or cohabiting, 85.5% were employed, 11.7% unemployed, and the rest had an unstable employment status at the time of the survey.

During the COVID-19 lockdown, the children in our sample spent about 3 h on average using screen-based devices for educational purposes (186.25 min) and 2 h 45 min for recreational purposes. Older children spent more time using screens (see Table 1 and Table 2). Only 42.5% of children did not exceed the recommended 2 h of recreational screen time during the lockdown.

Approximately one–third (34.3%) of the participants were engaged in physical activity lasting for 31–60 min and one-third (32.0%) were physically active for more than 1 h per day. The rest of the children did not meet the recommended guidelines for engaging in physical activity for 60 min per day. A quarter of the children (25.5%) spent less than 30 min outdoors in the springtime during the lockdown. There were no gender differences in screen use time for education (Mann–Whitney U = 11,208.00, *p* = 0.554) or leisure (Mann–Whitney U = 11,372.00, *p* = 0.705), as well as in physical activity (Mann–Whitney U = 11,477.00, *p* = 0.800) and time spent outdoors (Mann–Whitney U = 10,920.00, *p* = 0.303).

According to parental reports, the most frequent somatic symptoms in children during the lockdown were headaches (46.7% reported that their children experienced them often or sometimes) and stomach pains (44.4%). Children who suffered from diarrhea were significantly less physically active during the lockdown (Mann–Whitney U = 5982.00, *p* = 0.037). Children with headaches spent significantly more time using screens for educational purposes (Mann–Whitney U = 9160, *p* = 0.001) and were younger (Mann–Whitney U = 8454, *p* < 0.000). There were no significant gender differences in the prevalence of somatic symptoms.

One-third of parents (31.4%) reported that their children’s emotional well-being/behavior decreased during the lockdown. A total of 45.8% of the children experienced no change and the emotional well-being/behavior of the rest (22.9%) was viewed as improved. The children’s gender was not related to changes in emotional well-being/behavior (χ^2^ = 2.64, *p* = 0.268).

The correlational analysis of the study variables showed that screen time for educational purposes was negatively related to the level of children’s physical activity and time spent outdoors and positively related to the children’s age and the number of somatic symptoms (Table 2). Longer screen time for leisure activities was also associated with less physical activity, less time outdoors, and children’s older age, but not with somatic symptoms. Importantly, lower-educated parents reported higher levels of recreational screen time for their children. Our results also indicate that younger children were more physically active and had fewer somatic complaints. In addition, parents with a higher level of distress were better educated and reported their children being less physically active, spending less time outdoors, and having more somatic complaints. Lastly, parents with a higher level of distress reported a negative change in emotional well-being and behavior of their children and children’s better emotional well-being was related to longer time outdoors. 

Next, we explored how the experience of stressful life events (COVID-19 was not included in this experience) was related to other study variables. The results indicate that children of parents who reported stressful events within the past year spent less time outdoors during the confinement (Mann–Whitney U = 4172.00, *p* =0.003). Yet, the experience of stressful life events was not related to screen time, PA, the prevalence of somatic symptoms, or a change in emotional well-being/behavior during the confinement.

In the next stage of the analysis, hierarchical linear regression models were developed to examine significant predictors of children’s somatic symptoms. Children’s age was entered into the first step and explained only 1.5% of the variance in somatic symptoms, which was not significant (see Table 3). In the second step, the variables of screen time for education and for leisure, physical activity, and time outdoors were entered, and the total variance explained increased to 4.6%. Furthermore, screen time for education was the only significant predictor of children’s somatic symptoms. In the third step, parental variables were added (parental education, distress, and stressful life events) and it was found that children’s somatic symptoms were significantly predicted by longer screen time for education and higher parental distress. The final model included the addition of interactions between parental distress, SB, and PA variables, whereby the total variance explained increased to 15.1%. This model showed that screen time for education and higher parental distress continued to significantly predict children’s somatic symptoms; yet another significant negative predictor of this dependent variable was the interaction between screen time for education and parental distress. The latter result suggests that the association between somatic symptoms and screen time for education decreased when parental distress decreased as well (since a higher score on the variable of parental distress indicated a lower level of such distress).

In the final stage of the analysis, multinomial logistic regression models were created to explore changes in the children’s emotional well-being/behavior as a categorical outcome variable. The first step of the analysis included the children’s age; in the second step, the variables of screen time, physical activity, and time outdoors were added. The third step included the addition of parental variables (education, distress, and stressful life events), and interactions between SB, PA, and parental distress were added in the final step.

The results presented in Table 4 show that in the final model, lower levels of parental distress increased (vs. decreased) children’s emotional well-being/behavior during the lockdown (OR = 1.13; 95% CI = 1.06–1.20). Parental distress also significantly predicted a decrease in children’s emotional well-being and behavior as opposed to predicting no changes in the dependent variable (OR = 1.15; 95% CI = 1.09–1.22) (see Table 4). The interactions between SB, PA, and parental distress were not significant in predicting changes in the children’s emotional well-being/behavior.

## 4. Discussion

The COVID-19 pandemic has triggered an array of emotional, social, and physical issues for the whole family system. The aim of this study was to explore the relationships between child sedentary behavior, physical activity, mental and physical health, and parental distress in a sample of typically developing Lithuanian children aged 6–14 years during the COVID-19 pandemic lockdown in March–June 2020. The sociodemographic characteristics of the parents in the sample (73.5% of parents had a university-type education) corresponded with the main tendencies of educational attainment in Lithuania. Tertiary attainment in Lithuania is higher than in other OECD (Organization for Economic Co-operation and Development) countries, e.g., in 2018, 56% of 25–34 year-olds were tertiary educated, 11 percentage points more than the OECD average [40]. A better understanding of these relationships could provide valuable knowledge about risk and protective factors for children in crisis-ridden situations.

First, we found that screen time for educational purposes was related to somatic symptoms, mostly headaches and stomachaches. Studies conducted before the pandemic also established an association between excessive use of electronic devices and the presence of headaches [41,42]. On the one hand, long-lasting stress during a pandemic negatively affects the autonomic nervous system and cortex, thus causing psychosomatic and somatic symptoms and illnesses; on the other hand, excessive screen time, even for educational purposes, leads to reduced recreational activities, time outdoors, and an overload of the visual system [42]. The higher prevalence of somatic symptoms during a pandemic may be the result of accumulated negative effects of both factors.

Second, our findings show that, contrary to the hypothesis, screen time for recreation/leisure was not related to somatic complaints or changes in emotional well-being and behavior in children during the confinement. The associations between excessive screen time and poor mental health in children and adolescents were well established prior to confinement [7,43]. Some recent studies have found that longer screen leisure time is related to elevations in negative affect during the COVID-19 pandemic [28,29,44]. It must be noted that, differently from many other studies, the participants in our study were younger and all the variables were parent-reported. Moreover, in a pandemic context, screens were widely used for online social interactions and communication and thus may not result in a negative effect on mental health. Furthermore, screen time could have a soothing (or entertaining) effect in the short term, although it could be related to emotional and behavioral problems in the long term.

Most importantly, parental distress emerged as the most significant risk factor for children’s somatic and mental health during the confinement and stood out from the other parental and child variables included in the analysis. This is comparable with the results of other studies before the pandemic [45] and during the pandemic [36] that report the association between parental mental health problems and children’s psychosocial adjustment. The study conducted by Spinelli et al. (2020) [34] in Italy found that parental stress significantly increased children’s psychological, emotional, and behavioral problems [34]. As children often express distress through somatic complaints [46], the findings of our investigation are in line with prior studies indicating positive correlations between child and maternal psychological distress [47], as well as between parental stress and child somatic complaints [48].

Interestingly, screen time (for both educational or recreational purposes) was not related to parental distress or stressful life events in our study. However, parents with a higher level of distress reported their children being less physically active, spending less time outdoors, and having more somatic complaints. In addition, parents who had experienced stressful events within the past year were more likely to state that their children spent less time outdoors. It could be suggested that parents who are more stressed find it more difficult to encourage their children to be more physically active and spend more time outdoors. A number of studies have found positive associations between the physical activity of parents and their children [49,50]. The effect of parental stress on children’s well-being could be explained by the lower responsiveness of the stressed parents to children’s physical and emotional needs [31] and less optimal parenting practices [51]. We also found that lower parental education was related to longer screen time for recreational purposes, as was documented in other studies prior to confinement (e.g., [9,52]). Moreover, parents with a higher level of distress were higher educated in our study. Higher-educated parents might experience more struggles to balance work, their children’s upbringing, personal life, and being more involved in their children’s distance learning during a pandemic.

Importantly, our results show that parental distress interacted significantly with screen time for education in predicting children’s somatic symptoms. This means that as the level of parental distress decreased, so did the association between children’s somatic symptoms and screen time for education. In other words, parental emotional well-being served as a protective factor mitigating the negative effect of excessive screen time on children’s somatic health. Some pre-confinement studies indicated excessive homework as a source of students’ stress [53]. It can also be assumed that children who spend more time in front of screens for education are higher achievers that are vulnerable to school-related stress [54] and have more stress-induced psychosomatic complaints. In addition, because of the high level of stress, parents may be not able to provide effective support for children and modulate their anxiety, and may even be a source of additional stress for children.

Lastly, the findings of our study suggest that, on average, children used screens for about 3 h per day for educational purposes and 2 h 45 min per day for recreational purposes during the lockdown. Summing up, these 6–14-year-old children spent around 6 h per day in front of screens. These findings are comparable to the results of other recent studies, which found a considerable increase in screen time in children of all ages during the COVID-19 pandemic (e.g., [11,23]). Many countries have established a more than double increase in screen time compared to that before the pandemic [36,55]. In our study, 57.5% of children exceeded the recommended 2 h of recreational screen time. This amount of screen time is higher compared to other countries during the COVID-19 pandemic (e.g., in a Chinese sample of older children (see [10]), only one-fourth exceeded the recommendations of child health authorities). Furthermore, one-third of the children in our study did not meet the recommended guidelines of 60 min of physical activity per day and a quarter of our participants spent less than 30 min outdoors in springtime during the lockdown, although the level of children’s physical activity during the confinement in spring 2020 remained similar to that documented prior to confinement (cf. [8]). Interestingly, it appears that Lithuanian children spent even more time outdoors in the confinement compared to the time prior to the pandemic (cf. [8]). This could be explained by the fact that during the confinement in March–May 2020, the weather was favorable for staying outdoors and there were no restrictions for time outdoors in Lithuania. The findings in other countries were somewhat mixed and revealed either a considerable decrease in the physical activity of children since the onset of the pandemic [11,18,22] or, vice versa, an increase in active time during the lockdown [23]. The latter findings were explained by suggesting that children had more recreational time to do sports and, according to the self-determination theory, were more focused on health issues. Moreover, we found a negative link between screen time and physical activity. Such associations are in line with findings from other studies [56].

### Limitations and Strengths

It must be noted that our study has several limitations. First, as the study is cross-sectional, we cannot conclusively determine the directional effects. For example, we cannot tell whether parents experience more distress because their children had more physical and mental health problems or vice versa. Second, we did not measure the screen time, the level of physical activity, and time outdoors of the same children before the confinement. This did not allow for a comparison of the study variables prior to and during the spring confinement in 2020. Despite findings that parents can make accurate estimates of their children’s SB and PA [57], diary methods for tracking screen use and observational studies of children’s behavior could be used as additional reliable and informative measures in future research. Fourth, the results of our study should be generalized with caution because of the relatively small and non-representative sample.

The research captured a short but very important time of children’s lives when they had to undergo sudden and unexpected changes in their everyday physical and social life. This study is one of the first that considered the impact of parental stress in investigating the effect of an increase in SB and a decrease in PA on children’s mental and physical health. Parental stress was identified as an important risk factor for their physical and psychosocial functioning. The second strength of this study was the separation of screen time for educational and leisure purposes in the context of COVID-19 and distance learning. It is important to note that both the physical and mental health of children were investigated.

## 5. Conclusions

Summing up the results of our study, the confinement period in spring 2020 in Lithuania was characterized by an increase in screen time and relatively unchanged physical activity in children (compared to the period prior to the pandemic). In this context, it was parental distress that had the largest effect on children’s physical and mental health.

The results of the study suggest several implications for mental health professionals and parents. First, we found support for the negative effect of prolonged screen time for educational purposes on children’s physical health. The use of a screen for learning purposes should be separated from its use for recreational purposes. However, even when a screen is used for learning purposes, screen hygiene should be practiced (e.g., breaks to avoid eye strain). It is important to pay special attention to children with somatic complaints who spend excessive amounts of screen time for educational purposes. Maintaining an optimal level of physical activity, time outdoors, and limiting screen time may be important for the health of children and adolescents in stressful times.

Second, the results of our study revealed the importance of the level of parental distress in analyzing children’s sedentary behavior and physical and mental health. Professionals and caretakers need to be aware of the severe situation and implement more effective interventions for parental support to minimize the negative impact of the COVID-19 pandemic on children’s and adolescents’ health. Parents should be more attentive to their mental health needs and follow appropriate self-care steps, as well as apply for professional help when needed. Pediatric healthcare professionals should be more mindful of parental emotional wellbeing while providing services for children during a pandemic situation. More research is needed to further investigate the mediating or moderating effect of parental mental health in the relationship between children’s sedentary behavior, physical activity, and health when facing the extraordinary situation of COVID-19 pandemic and the consequent physical and social restrictions. In the context of the ongoing pandemic situation, longitudinal monitoring of children’s health and measuring the long-term consequences of decreased SB and PA would provide more important insights. 

## Figures and Tables

**Table 1:** Baseline characteristics of the participants.

Characteristics	(*n* = 306)
Child age (years) ^a^	9.65 (SD = 1.94)
Child age	
% 6–10 years	71.6 (*n* = 219)
% 11–14 years	28.4 (*n* = 87)
Child gender	
% Girls	52.9 (*n* = 162)
% Boys	47.1 (*n* = 144)
Screen time for education (min) ^a^	186.25 (SD = 84.28)
Screen time for leisure (min) ^a^	165.31 (SD = 93.29)
% Up to 2 h of screen time for leisure	42.5 (*n* = 130)
% 2–4 h of screen time for leisure	45.4 (*n* = 139)
% 5 and more hours of screen time for leisure	12.1 (*n* = 37)
Physical activity	
% Almost not physically active at all	8.8 (*n* = 27)
% Less than 30 min	24.8 (*n* = 76)
% 31–60 min	34.3 (*n* = 105)
% More than 60 min	32.0 (*n* = 98)
Time outdoors	
% Almost no time outdoors	8.5 (*n* = 26)
% Less than 30 min	17.0 (*n* = 52)
% 31–60 min	28.4 (*n* = 87)
% More than 60 min	46.1 (*n* = 141)
Somatic symptoms: nausea	
% No	84.0 (*n* = 257)
% Sometimes/often	49.0 (*n* = 16)
Somatic symptoms: constipation	
% No	83.7 (*n* = 256)
% Sometimes/often	16.3 (*n* = 50)
Somatic symptoms: diarrhea	
% No	81.0 (*n* = 248)
% Sometimes/often	19.0 (*n* = 58)
Somatic symptoms: stomach pains	
% No	55.6 (*n* = 170)
% Sometimes/often	44.4 (*n* = 136)
Somatic symptoms: headaches	
% No	53.3 (*n* = 163)
% Sometimes/often	46.7 (*n* = 143)
Other somatic symptoms	
% No	80.4 (*n* = 246)
% Sometimes/often	19.6 (*n* = 60)
Parental education	
% Low (≤12 years)	13.4 (*n* = 41)
% Medium (13–15 years)	9.2 (*n* = 28)
% High (≥16 years)	73.5 (*n* = 225)
% Not specified	3.9 (*n* = 12)
Stressful life events	
% Yes	14.1 (*n* = 43)
% No	85.9 (*n* = 263)
Change in the child’s emotional well-being/behavior during lockdown	
% Decreased	31.4 (*n* = 96)
% No change	45.8 (*n* = 140)
% Increased	22.9 (*n* = 70)

Note: ^a^ Values are presented in means and standard deviations (SD).

**Table 2:** Bivariate correlations among study variables.

Variable	1	2	3	4	5	6	7	8	9
1. Screen time for education	-								
2. Screen time for leisure	0.11	-							
3. Physical activity	−0.16 **	−0.21 **	-						
4. Time outdoors	−0.16 **	−0.22 **	0.66 **	-					
5. Child’s somatic symptoms	0.12 *	−0.01	−0.10	−0.03	-				
6. Child’s emotional well-being/behavior	−0.02	−0.06	0.09	0.13 *	−0.06	-			
7. Child’s age	0.41 **	0.19 **	−0.22 **	−0.09	0.12 *	0.05	-		
8. Parental education	−0.11	−0.21 **	−0.05	−0.11	0.05	−0.03	−0.14 *	-	
9. Parental distress	0.03	0.01	−0.17 **	−0.17 **	0.23 **	−0.27 **	−0.10	0.14 *	-

Note: * *p* < 0.05, ** *p* < 0.01.

**Table 3:** Hierarchical linear regression models predicting children’s somatic symptoms.

Predictors	*β*	SE	*p*	R^2^	R^2^ Change	F	*p*
Step 1: Child’s age							
Child’s age	0.12	0.06	0.033	0.015	0.015	4.57	0.033
Step 2: Child’s age, SB, and PA							
Child’s age	0.04	0.07	0.502	0.046	0.031	2.78	0.018
Screen time for education	**0.17**	**0.00**	**0.009**				
Screen time for leisure	−0.04	0.00	0.521				
Physical activity	−0.12	0.16	0.126				
Time outdoors	0.11	0.16	0.163				
Step 3: Child’s age, SB, PA, and parental variables							
Child’s age	0.09	0.07	0.172	0.122	0.076	4.94	<0.000
Screen time for education	**0.16**	**0.00**	**0.011**				
Screen time for leisure	−0.02	0.00	0.797				
Physical activity	−0.08	0.16	0.309				
Time outdoors	0.14	0.16	0.070				
Parental education	0.07	0.16	0.256				
Parental distress	**0.27**	**0.02**	**0.000**				
Stressful life events (0—no; 1—yes)	−0.03	0.33	0.658				
Step 4: Child’s age, SB, PA, parental variables, and interactions							
Child’s age	0.08	0.07	0.215	0.151	0.151	4.17	<0.000
Screen time for education (STE)	**0.15**	**0.00**	**0.018**				
Screen time for leisure (STL)	−0.01	0.00	0.919				
Physical activity (PA)	−0.06	0.16	0.468				
Time outdoors (TO)	0.11	0.17	0.192				
Parental education	0.04	0.16	0.458				
Parental distress	**0.27**	**0.02**	**<** **0.000**				
Stressful life events	−0.03	0.32	0.612				
STE × parental distress	**0.16**	**0.11**	**0.005**				
STL × parental distress	−0.07	0.12	0.240				
PA × parental distress	0.02	0.15	0.813				
TO × parental distress	0.05	0.15	0.542				

Note: SB—Sedentary behavior, PA—Physical Activity. Bold text indicates a statistically significant difference with a *p*-value less than 0.05.

**Table 4:** Multinomial regression models predicting changes in children’s emotional well-being/behavior.

Predictors	Change in Emotional Well-Being/Behavior					
	Increased vs. Decreased		No Change vs. Decreased		Increased vs. No Change	
	B (SE)	OR [95% CI]	B (SE)	OR [95% CI]	B (SE)	OR [95% CI]
Step 1: Child’s age						
Child’s age	0.08 (0.08)	1.09 [0.93, 1.28]	0.09 (0.07)	1.09 [0.95, 1.25]	0.00 (0.08)	1.00 [0.86, 1.16]
Step 2: Child’s age, SB, and PA						
Child’s age	0.12 (0.10)	0.12 [0.93, 1.35]	0.16 (0.08) *	**1.18 [1.01, 1.38]**	−0.05 (0.09)	0.95 [0.80, 1.13]
Screen time for education	0.00 (0.00)	1.00 [0.996, 1.004]	0.00 (0.00)	1.00 [0.994, 1.001]	0.00 (0.00)	1.00 [0.998, 1.006]
Screen time for leisure	0.00 (0.00)	1.00 [0.996, 1.004]	0.00 (0.00)	1.00 [0.995, 1.001]	0.00 (0.00)	1.00 [0.998, 1.004]
Physical activity	0.07 (0.23)	1.07 [0.68, 1.69]	0.07 (0.20)	1.07 [0.73, 1.58]	0.00 (0.21)	1.00 [0.66, 1.52]
Time outdoors	0.30 (0.23)	1.35 [0.86, 2.11]	0.16 (0.19)	1.17 [0.81, 1.69]	0.14 (0.21)	1.15 [0.76, 1.75]
Step 3: Child’s age, SB, PA, and parental variables						
Child’s age	0.11 (0.10)	1.12 [0.92, 1.36]	0.15 (0.09)	1.16 [0.98, 1.38]	−0.04 (0.09)	0.96 [0.80, 1.15]
Screen time for education	0.00 (0.00)	1.00 [0.996, 1.005]	0.00 (0.00)	1.00 [0.994, 1.001]	0.00 (0.00)	1.00 [0.999, 1.007]
Screen time for leisure	0.00 (0.00)	1.00 [0.995, 1.003]	0.00 (0.00)	1.00 [0.994, 1.001]	0.00 (0.00)	1.00 [0.998, 1.005]
Physical activity	0.12 (0.24)	1.02 [0.63, 1.64]	−0.01 (0.21)	0.99 [0.66, 1.49]	0.03 (0.22)	1.03 [0.66, 1.59]
Time outdoors	0.23 (0.24)	1.25 [0.78, 2.02]	0.08 (0.20)	1.08 [0.73, 1.61]	0.15 (0.23)	0.98 [0.93, 1.04]
Parental education	−0.12 (0.25)	0.87 [0.54, 1.46]	−0.13 (0.22)	0.88 [0.57, 1.36]	0.01 (0.22)	1.01 [0.66, 1.55]
Parental distress	**−0.12 (0.** 03) ***	**1.12 [ 1.06, 1.19]**	**−0.13 (0** .03) ***	**1.14 [1.09, 1.21]**	0.02 (0.03)	0.98 [0.93, 1.04]
Stressful life events (0—no stressful life events)	−0.44 (0.47)	0.64 [0.25, 1.63]	−0.11 (0.44)	0.90 [0.3, 2.11]	−0.34 (0.44)	0.72 [0.30, 1.70]
Step 4: Child’s age, SB, PA, parental variables, and interactions						
Child’s age	0.11 (0.10)	1.11 [0.91, 1.36]	0.14 (0.90)	1.15 [0.97, 1.36]	−0.03 (0.09)	0.97 [0.81, 1.16]
Screen time for (STE)	0.00 (0.00)	1.00 [0.995, 1.004]	0.00 (0.00)	1.00 [0.993, 1.001]	0.00 (0.00)	1.00 [0.998, 1.007]
Screen time for leisure (STL)	0.00 (0.00)	1.00 [0.995, 1.003]	0.00 (0.00)	1.00 [0.994, 1.001]	0.00 (0.00)	1.00 [0.998, 1.005]
Physical activity (PA)	0.06 (0.26)	1.06 [0.64, 1.77]	0.09 (0.23)	1.09 [0.70, 1.70]	−0.03 (0.23)	0.97 [0.62, 1.52]
Time outdoors (TO)	0.13 (0.27)	1.14 [0.66, 1.94]	−0.04 (0.24)	0.96 [0.61, 1.53]	0.17 (0.24)	1.18 [0.74, 1.89]
Parental education	−0.09 (0.26)	0.91 [0.55, 1.52]	−0.20 (0.23)	0.82 [0.53, 1.28]	0.11 (0.23)	1.11 [0.72, 1.74]
Parental distress (PD)	−0.12 (0.03) ***	**1.13 [1.06, 1.20]**	−0.14 (0.03) ***	**1.15 [1.09, 1.22]**	0.02 (0.03)	0.98 [0.92, 1.04]
Stressful life events (0—no stressful life events)	−0.47 (0.47)	0.63 [0.25, 1.58]	−0.07 (0.44)	0.96 [0.40, 2.28]	−0.43 (0.45)	0.65 [0.27, 1.58]
STE × PD	0.17 (0.18)	0.84 [0.59, 1.20]	0.11 (0.16)	0.90 [0.66, 1.22]	0.07 (0.17)	0.94 [0.67, 1.31]
STL × PD	−0.08 (0.19)	1.08 [0.75, 1.56]	0.17 (0.16)	0.87 [0.63, 1.19]	−0.22 (0.17)	1.24 [0.88, 1.75]
PA × PD	−0.17 (0.23)	1.19 [0.75, 1.87]	−0.15 (0.21)	1.56 [0.76, 1.75]	−0.03 (0.22)	1.03 [0.67, 1.57]
TO × PD	−0.07 (0.25)	1.07 [0.66, 1.76]	0.37 (0.23)	0.69 [0.44, 1.08]	−0.44 (0.24)	1.56 [0.98, 2.48]

Notes: * *p* < 0.05, *** *p* < 0.001; the percentage of correctly classified cases in the final model was 53.1%, the Nagelkerke R^2^ = 0.192. OR—Odds ratio, CI—Confidence interval, SE—Standard error. Bold text indicates a statistically significant difference with a *p*-value less than 0.05.

## Data Availability

The datasets are available on reasonable request. If anyone wants to get access to the data, please send an email to rima.breidokiene@fsf.vu.lt.
